# The N2pc Is Increased by Perceptual Learning but Is Unnecessary for the Transfer of Learning

**DOI:** 10.1371/journal.pone.0034826

**Published:** 2012-04-02

**Authors:** An An, Meirong Sun, Yun Wang, Fang Wang, Yulong Ding, Yan Song

**Affiliations:** 1 State Key Laboratory of Cognitive Neuroscience and Learning, Beijing Normal University, Beijing, China; 2 Department of Psychology, Sun Yat-Sen University, Guangzhou, China; Ecole Polytechnique Federale de Lausanne, Switzerland

## Abstract

**Background:**

Practice improves human performance in many psychophysical paradigms. This kind of improvement is thought to be the evidence of human brain plasticity. However, the changes that occur in the brain are not fully understood.

**Methodology/Principal Findings:**

The N2pc component has previously been associated with visuo-spatial attention. In this study, we used event-related potentials (ERPs) to investigate whether the N2pc component changed during long-term visual perceptual learning. Thirteen subjects completed several days of training in an orientation discrimination task, and were given a final test 30 days later. The results showed that behavioral thresholds significantly decreased across training sessions, and this decrement was also present in the untrained visual field. ERPs showed training significantly increased the N2pc amplitude, and this effect could be maintained for up to 30 days. However, the increase in N2pc was specific to the trained visual field.

**Conclusion/Significance:**

Training caused spatial attention to be increasingly focused on the target positions. However, this process was not transferrable from the trained to the untrained visual field, which suggests that the increase in N2pc may be unnecessary for behavioral improvements in the untrained visual field.

## Introduction

The perceptual performance of human beings can be improved through repeated training. During the past decades, this improvement has been found in a variety of visual tasks, such as texture discrimination [1], motion direction [2], [3], spatial phase [4], hyperacuity [5] orientation discrimination [6] and visual search [7], [8], [9], [10]. From these earlier studies, various theories with different emphases have been proposed to explain the process of perceptual learning., Some have suggested that learning-induced improvement may occur at the low level of the visual cortex in the adult human brain, because many behavioral studies show that learning-induced improvements are specific to the stimulus attributes, such as orientation [1], [11], [5], [12] and location [13]. This dominant assumption was strongly supported by human neuroimaging studies. One example is the training of visual texture discrimination leading to increases in the activity of the corresponding quadrant of the visual field representation in V1 [14]. On the other hand, some psychophysical studies have argued that stimulus specificity does not hold all the time. Learning seems to be specific to the task used during the training and for the visual context [15], which suggests that perceptual learning might occur at a higher level than V1/V2. Also, some researchers found that perceptual learning might be influenced by some high-level mechanisms, such as attention [16] and decision making [17].

In recent years, more and more researchers have found that attention is essential in perceptual learning [18], [19], [20]. However, a few studies have shown that perceptual learning occurred even for sub-threshold task-irrelevant stimuli [21], [22]. These results indicated that attention might not be necessary for perceptual learning under some conditions. Therefore, the effect of attention in perceptual learning is still controversial.

In the current study, we used event-related potentials (ERP) as a tool to explore how spatial attention changes during perceptual learning. Previous studies found that many ERP components, which may be related to attention, changed as part of the learning process. For instance, in a classical orientation discrimination learning task, Song et al. (2010) [23] found that along with the behavioral improvement, the N1 and N2 constantly decreased, while the P2 or P3 increased.

In addition to these classical components in ERP studies, there is another important component named N2pc, which has been suggested to have a stronger relationship with attention. The N2pc component is a difference waveform of N2 and is a negative-going waveform in the time window between 200 and 300 ms. It is typically observed at posterior scalp sites contralateral to the location of the target, so the N2pc was obtained by subtracting the ipsilateral N2 from the contralateral one. Previous studies have showed that this component reflects the focusing of attention on a target and can be influenced by task difficulty, which in part refers to the amount of spatial attention required in a task. [24]. Because of the strong relationship between N2pc and spatial attention, it has become a useful tool in the study of visual–spatial attention, especially when used to investigate the visual search task [25].

Although many researchers have used N2pc to explore the role of attention, little has been done to investigate the changes of N2pc during perceptual learning. Recently, Hamamé et al. (2011) [26] published the first article investigating the relationship between N2pc and perceptual learning. They found that N2pc was significantly increased during learning. Moreover, the N2pc increase was specific to the stimulus orientation and correlated with the behavioral performance. In the current study, we further tested whether learning a visual search task could modify the amplitude of N2pc, whether the N2pc change was specific to stimulus location, and whether the change in N2pc was temporary or longer-lasting.

## Results

### Psychological data

**Figure 1:**
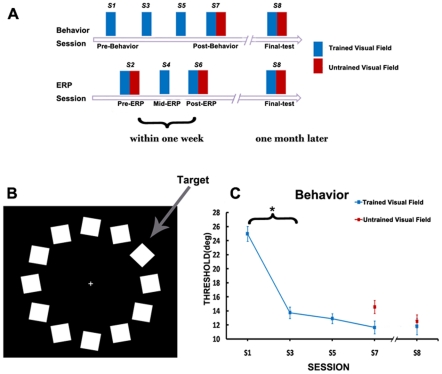
Procedure, stimulus and behavioral results. (A) Illustration of the whole experiment procedure. (B) An example of the stimulus used in the present experiment. For each trial the search array contained a target on the left or right visual field. (C) The group mean threshold with standard errors in each session. The 5 original scores for the trained visual field (S1, S3, S5, S7 and S8) for each subject were normalized by subtracting a subject deviation score consisting of that subject's mean (across 5 blocks) minus the grand mean (across participants and blocks).And the same calculation also used in the untrained visual field.

Figure 1C showed the mean thresholds in the trained and untrained visual field during the whole behavioral sessions. As expected, several days of training greatly increased the subject's discriminability. For the trained visual field, thresholds decreased from 25.3°in S1 to 12.1°in S7 (Repeated Measures ANOVA, F_3,11_ = 21.7, P<0.001) and the significant decrease was observed only between S1 and S3 (t_13_ = 7.83, P<0.001). When examining the S7 data alone, we found that there were no significant differences between the thresholds in the trained and untrained visual field (t_13_ = −1.97, p = 0.07), suggesting that the performance improvement transferred almost completely from the trained to the untrained visual field after long-term training (the percent of transfer is 83.1%). One month later, the thresholds in S8 still did not show significant change (S7 vs. S8: trained visual field: t_8_ = 0.794, p = 0.450; untrained visual field: t_8_ = 2.191, p = 0.06), indicating that the behavioral improvement was successfully preserved for both visual fields even after one month. Finally, we counted the accuracy of the catch trial in which the fixation cross was rotated. The result of 95% accuracy indicated that the subjects focused well on the cross throughout the whole experiment.

### Electrophysiological data: ERP waves

**Figure 2:**
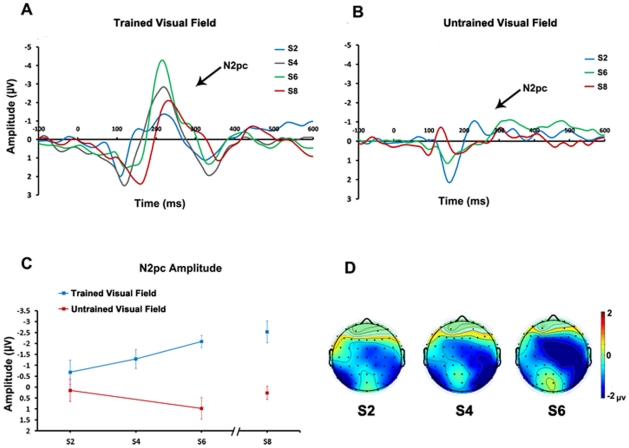
The N2pc amplitude of trained/untrained visual fields and topographic distribution. (A) Shows the group-mean ERPs of trained visual field in 4 ERP sessions. The part under shadow is the N2pc time window we used in analysis. (B) Shows the group-mean ERPs of untrained visual field in the pre and post ERP sessions. The shadow part stands for the N2pc time window. (C) Illustrates the N2pc change for both trained and untrained visual fields. We also normalized the ERP data in the same way as we did to behavioral data. (D)Takes left training visual field as an example to see the topographic distribution of scalp potential during the N2pc time-window from S2 to S6.

As shown in Figure 2A and 2B, there was a significant training effect on N2pc amplitude. More importantly, this effect was different between the trained and untrained visual field (Repeated Measures ANOVA, main effect of training (S2, S4 vs. S6): F_2,11_ = 5.878, p = 0.018; training (S2 vs. S6) × location (trained vs. untrained visual field): F_1,12_ = 5.614, p = 0.035). Specifically, training in the trained visual field significantly increased the N2pc amplitudes, and the N2pc increase did not transfer to the untrained visual field. Taking the target shown in the left visual field as an example, we can clearly see that the scalp potential in the contralateral hemisphere (right hemisphere) became more negative than the ipsilateral hemisphere (left hemisphere) after 3 training sessions (Figure 2D).

Further analysis showed that for the trained visual field, the N2pc amplitude in S6 was significantly larger than that in S2 (t_12_ = 3.029, p = 0.010) but had no significant difference when comparing to that in S8 (t_8_ = 0.578, p = 0.579). Also the amplitude in S8 was significantly different with the one in S2 (t_8_ = 2.524, p = 0.036), indicating that the learning effect associated with the N2pc change is preserved even after 30 days. However, when comparing the data of the untrained visual field between S2 and S6, there was no significant difference (t_12_ = −1.099, p = 0.293), and one month later, the same non-significant result also appeared between S2 and S8 (t_8_ = −1.362, p = 0.210), which meant that the N2pc increment did not transfer from the trained to untrained visual field both during the training and after 30 days.

As mentioned above, the subjects were only presented with the targets in the trained visual field in S1. Did the behavioral pre-test in S1 influence the ERP pre-test data in S2? To strictly exclude any possible confounding factors related to the N2pc, we next examined the N2pc amplitude between the trained and untrained visual field in S2. The results showed no significant difference between the two visual fields in the ERP pre-test (t_12_ = −0.546, p = 0.595), indicating that the learning effect associated with the N2pc change was induced by the later training, but not the behavioral pre-test or initial ERP difference.

Although the accuracy of detecting the fixation rotation has been more than 95%, we still examined whether eye movement had some effect on the current results, since the EOG had been very difficult to control for some subjects and may have influenced the final results. A planned comparison was conducted on the EOG differences during the period of the N2pc (about 270∼310 ms after stimulus, different between subjects), or immediately before it (180∼220 ms after stimulus). The result showed no differences between pre-learning and post-learning in both VEOG and HEOG. The EOG amplitudes did not differ as a function of learning, suggesting that the EOG differences during these conditions cannot account for the final results.

We also analyzed if there is any relationship between training-induced changes in the N2pc amplitude and the psychological thresholds. However, no reliable correlation was found between behavioral improvement and ERP changes. Because of large individual differences among the subjects, we also reran the ANOVA using the normalized data. This analysis still revealed a non-significant correlation.

Finally, we investigated the peak latency of the N2pc during the long-term training. The results showed no reliable differences among the ERP sessions for both the trained and untrained visual fields.

## Discussion

### Behavioral improvement

Using the behavioral threshold as an index, this study showed that several days of training on a visual search task resulted in an improvement (the percent of improvement is 52.2%) in behavioral performance, and the improvement was largely preserved after 30 days. This learning effect is consistent with previous studies using threshold or accuracy as a measure, supporting the prevailing notion that training would induce a constant, long-term change in human perceptual performance. However, we unexpectedly found that the improvement in the trained visual field has nearly completely transferred to the untrained side (the percent of transfer is 83.1%), inconsistent with some prior behavioral studies. For instance, Schoups et al. (1995) [5] found that orientation discrimination learning is precisely specific to the trained retinal location, even if the location is merely 2.5°away from the trained one. This discrepancy between these findings might result from the differences in the stimuli, task and training procedure. On the other hand, recent psychophysical studies suggest that the specificity might in fact not be inherent in perceptual learning and could be influenced by several other factors. First, Aberg et al. (2009) [27] found that the intensity of training can strongly influence learning efficiency and occurrence of transfer. That is the transfer of learning depended on the number of trials within a session, even though the overall amount of training was identical. Only the training under intermediate intensity conditions (about 400 trails per session) could induce the transfer of learning. In our study, each session consists of at least 200 trials and not more than 300 trails, in which the training intensity is just within the intermediate range according to Aberg et al.'s study. Second, another study of Zhang et al. (2010) [28] found that a brief pretest in the untrained location would cause a complete transfer of learning from the trained to untrained location in an orientation discrimination learning. Tartaglia et al., (2009) [29] further demonstrated that the pretest itself cannot induce the transfer of learning. It might be the pretest (in the untrained condition) and the training (in the trained condition) together that promote the transfer of learning. In the present study, we first determined the ERPs in the trained and untrained visual fields in S2. Subsequently; observers were trained with intermediate training intensity, with the targets in the trained visual field. Therefore, the transfer in the current study was reasonable according to several recent psychophysical studies.

### ERP changes

#### 1. Training

Along with the reduction of behavioral thresholds, the N2pc amplitude became larger on the trained visual field, and this change did not occur on the peak latency. Many previous studies on perceptual learning have detected the similar changes in a few classic ERP components after training. For example, Qu et al. (2010) [30] has found that N1 and P2 became larger after training on a visual search task and that the peak latency was not changed even after six months. This result suggested that the ERP amplitudes were more sensitive to training than ERP latency. Moreover, the increase in N2pc in our experiment was consistent with a recent ERP study, in which Hamamé and his colleagues (2011) [26] found that the N2pc amplitude increased significantly after training on an orientation discrimination task.

Previous studies have suggested that N2pc has a close relationship with attention. For instance, Eimer (1996) [24] found that N2pc reflects the process of focusing attention on a target and that N2pc amplitude could be influenced by the task difficulty. Furthermore, this kind of attention related to N2pc is generally interpreted as the attentional filtering process, and N2pc is thought to be the electrocortical correlate of the distractor-suppression mechanism [9], [31]. In our study, we suggest that the increase in N2pc may reflect the brain focusing more spatial attention on the specific positions. At the beginning of the experiment, subjects divided most of their attention evenly on the items in the attended visual field. During several practice sessions, they learned to focus more of their limited attention at target positions, and thus less attention at other, irrelevant positions. This focusing can be considered as one kind of distractor suppression that improves the efficiency of the detection process.

There was a possibility that the change of N2pc may be a temporary phenomenon, with no relationship with long-term learning. However, the final test in the present study showed that the N2pc increase was successfully preserved after one month. These results dispel the notion that the changes during our experiment may be the result of adaptation.

#### 2. Specificity

Interestingly, we found that the N2pc amplitude of the untrained visual field was not significantly changed during the seven successive sessions and remained unchanged even one month later. Based on this result, we suggest that the ability to focus spatial attention does not transfer to the untrained visual field.

Based on this lack of transfer between the two visual fields, how can we explain the behavioral improvement in the untrained field? One explanation is that the subjects learned skills which assisted them to in making the right response. Recently, an increasing number of researches have indicated that a single cortical area or process is unlikely to be responsible for perceptual learning, and that perceptual learning is a refinement of synergistic processes in multiple stages and cortical areas, including those dedicated to sensory processing, engaged in top-down control, and those involved in working memory and decision making [32]. For instance, a recent fMRI study showed both the retinotopic early visual cortex and nonretinotopic higher brain areas related to decision making are involved in visual discrimination and perceptual learning [17]. In the present study, decision making or other high level functions may explain the improvement of performance in the untrained visual field.

In the current study, we used ERP technology to explore the change of visual spatial attention during orientation discrimination learning and the relationship between attention, perceptual learning and its location specificity. The behavioral thresholds were significantly decreased after several days of training, and this decrease transferred completely to the untrained visual field. Moreover, the change in thresholds could be preserved for one month in both visual fields. Interestingly, the N2pc component, which has been generally connected with visual-spatial attention, changed simultaneously with the behavioral performance improvement in the trained visual field, but remained unchanged in the untrained visual field throughout the long-term training. Taken together, this result suggested that training could make visual–spatial attention increasingly focused on the target positions, thus enhancing the efficiency of target detection in the trained locations. However, this process did not transfer from the trained to untrained visual field, which suggests that it may be not necessary for the behavioral improvement in the untrained visual field to occur. Further studies need to be performed in order to clarify how signals in higher levels and visual–spatial attention interact with each other to promote visual perceptual learning.

## Materials and Methods

### Ethics Statement

All experimental procedures were approved by the Beijing Normal University Institutional Review Board. Research was conducted according to the principles expressed in the Declaration of Helsinki and the experiments were undertaken with the understanding and written consent of each participant. All experiments were performed at the State Key Laboratory of Cognitive Neuroscience and Learning of the University.

### Subjects

Thirteen undergraduate and graduate students (three male and ten female) between 21 and 26 years (mean age: 22) participated in this experiment as paid volunteers. All subjects had normal or corrected-to-normal vision and were right-handed. None of them had ever participated in similar tasks. Informed consent was obtained from each subject before the experiment.

### Stimuli

The stimuli used in the experiment are illustrated in Figure 1B. In each trial, the gray fixation cross was first presented on the screen for the first 500 ms, and then the whole circular search array (5°×5°) was presented for 200 ms. The circular search array consists of 12 squares (1.7°×1.7°) positioned along the visual circle at a distance of 5° visual angle from the fixation cross. After this 200 ms period, the squares disappeared with the fixation cross still showing on the screen, waiting for the subjects' response. After the response, there was a blank lasting between 200 to 400 ms randomly. All fixation crosses and squares were gray (half contrast) on a black background.

Among 12 squares, there was only one square (target) whose diagonals were horizontal or vertical, while the other squares (distractors) had diagonals that were oblique. The target was randomly presented at either the right visual field (2 or 4 o'clock position) or left visual field (8 or 10 o'clock position). For each subject, one visual field was selected for training (left or right, counterbalanced across subjects). The selected visual field was named “the trained visual field”, while the other visual field was named “the untrained visual field”. In each session, subjects were instructed to pay attention to only one visual field (trained or untrained) and then reported the target's position by pressing different keys. In 5% of trials, the fixation was rotated 45° when the search array appeared. This was done to control eye movement. During these special trials, the subject only needed to press another key to report the detection of the fixation rotation. Subjects were seated in a dimly lit, sound attenuated and electrically shielded cabin to view the stimuli on a computer monitor from a distance of 1000 cm.

### Procedure

Subjects completed the experiment in seven successive days (Figure 1A), including four behavioral sessions in S1, S3, S5, S7 and three ERP sessions in S2, S4, S6. One month later, nine subjects were invited back and performed a final test in S8.

All behavioral sessions were conducted using the staircase procedure, and auditory feedback was given on incorrect responses. The staircases followed the three-down, one-up staircase rule, meaning the included angle (from 0° to 45° between the distractor and target diagonals decreased after 3 successive correct responses and increased after 1 incorrect response). This resulted in a 79.4% convergence rate. Each staircase consisted of four preliminary reversals and six experimental reversals. The step size of the staircase was 1°. The geometric mean of the experimental reversals was taken as the threshold for each staircase run.

In S1 (behavioral pre-test), subjects were tested for the initial threshold in the trained visual field. In S3 and S5 (behavioral training sessions), we further trained the subject in only the trained visual field. In S7 (behavioral post-test), both visual fields were tested to investigate if the learning effects transferred from the trained to untrained visual field. There were five or six staircases (about thirty minutes) in the behavioral pre-test and post-test sessions for each visual field and ten (about one hour) in the behavioral training sessions.

In the ERP sessions, the included angle between target and distractors was fixed for each subject (the angle was acquired in the behavioral pre-test in S1). In S2 (pre-ERP) and S6 (post-ERP), the EEG signals for both the trained and untrained visual fields were recorded. in S4 (Mid-ERP), only the EEG for the trained visual field was recorded. In S8 (final test), we tested the subjects' behavioral thresholds and EEG for both visual fields. For each visual field, there were 200 trials in 4 blocks. Each block required 2 to 3 minutes. All ERP sessions were conducted using the E-prime software.

### EEG recording

When the subjects took part in the ERP sessions, their Electroencephalogram (EEG) was recorded using a SynAmps EEG amplifier and the Scan 4.2 package (NeuroScan, Inc.). A Quick-cap with 62 tin scalp electrodes was used. Horizontal and vertical electro-oculograms (EOGs) were also recorded. The EEG was physically referenced to the left mastoid and then was off-line re-referenced to the average of the left and right mastoid. Electrode impedance was kept below 5 kΩ. The EEG was amplified with a band pass of 0.1 to 40 Hz, digitized on-line at a sampling rate of 500 Hz. Each epoch of EEG was 200 ms of pre-stimulus to 600 ms of post-stimulus. The EEG for all stimuli within each session was averaged. Trials contaminated by eye blinks, eye movement, or muscle potentials exceeding ±60 µV at any electrode as well as incorrect behavioral responses were excluded from the ERP averages, resulting in exclusion of about 10% of the trials from the average. Therefore, there were about 180 stimulus-related EEG segments averaged for each session. The baseline for ERP measurements was the mean voltage of a 200 ms pre-stimulus interval.

### Data Analysis

For the psychophysical data, the thresholds were analyzed. For ERP data, the effects of training were studied by examining changes in amplitudes of N2pc on the P5/P6 electrodes. For each subject, the N2pc amplitude was calculated as the mean value of a 50-ms window centered at the average peak latency at each electrode.

Repeated measures ANOVA with the factors ‘training’ and ‘location’ (trained vs. untrained visual field) and two tailed paired sample t-tests were used to analyze the training effects, as well as their preservations. Significance levels of the *F* ratios were adjusted with the Greenhouse-Geisser correction. All the analyses were conducted with original data.
